# Resistance screening and trend analysis of imported *falciparum* malaria in NSW, Australia (2010 to 2016)

**DOI:** 10.1371/journal.pone.0197369

**Published:** 2018-05-29

**Authors:** Christiane Prosser, Wieland Meyer, John Ellis, Rogan Lee

**Affiliations:** 1 Molecular Mycology Research Laboratory, Centre for Infectious Diseases and Microbiology, Westmead Clinical School, Faculty of Medicine and Health, Marie Bashir Institute for Infectious Diseases and Biosecurity, The University of Sydney, Sydney, New South Wales, Australia; 2 School of Life Sciences, University of Technology Sydney, Sydney, New South Wales, Australia; 3 Westmead Institute for Medical Research, Westmead, New South Wales, Australia; 4 Centre for Infectious Diseases and Microbiology Laboratory Services, ICPMR, Westmead Hospital, Westmead, New South Wales, Australia; Université Pierre et Marie Curie, FRANCE

## Abstract

**Background:**

The World Health Organization currently recommends artemisinin (along with a partner drug) as the global frontline treatment for *Plasmodium falciparum* malaria. Artemisinin resistant *P*. *falciparum* are now found throughout the greater Mekong subregion of South East Asia. Several polymorphisms in the parasite’s kelch gene have been demonstrated to confer artemisinin resistance. While genotypes within the greater Mekong subregion are thoroughly examined in the literature, *P*. *falciparum* populations within several areas that do not (yet) have endemic resistance are underrepresented.

**Results:**

This investigation characterised the *Pf*kelch13 propeller domains from 153 blood samples of 140 imported cases of *P*. *falciparum* malaria in New South Wales from 2010 to 2016. A low level of propeller domain diversity was observed, including the C580**Y** coding mutation most strongly associated with artemisinin resistance in South East Asia. The resistance genotype was found in a sample originating in Papua New Guinea, where this mutation, or artemisinin treatment failure, have not been previously reported. Sequencing a panel of geographically informative polymorphisms within the organellar genomes identified the C580**Y** parasite as having Oceanic origins. Patient data analysis revealed that New South Wales, Australia, *P*. *falciparum* malaria cases often originated from regions with limited drug resistance screening.

**Conclusions:**

The C580**Y** finding from outside of the greater Mekong subregion supports the consensus to upscale molecular surveillance of artemisinin resistance outside of South East Asia. The genetic screening results identify a risk of importing resistant *falciparum* malaria to Australia, supporting an ongoing surveillance protocol to pre-empt treatment failure and contribute to global data gathering.

## Introduction

The global frontline treatment for *Plasmodium falciparum* malaria is Artemisinin Combination Therapy (ACT) [1]. In the absence of an alternative frontline therapy, it is of utmost importance ACT efficacy is preserved. Artemisinin treatment failure was first observed on the Thai-Cambodian border and has since spread throughout the greater Mekong subregion of South East Asia [2]. Artemisinin resistance is characterised by a parasite clearance half-life of >5hrs, although ACT resistance needs to be considered together with sensitivity to the ACT partner drug [3]. Landmark studies conducted in 2008 by Noedl *et al*. and 2009 by Dondorp *et al*. first reported artemisinin resistance [4, 5]. More recently, resistance is described regarding known molecular markers. Several Single Nucleotide Polymorphisms (SNPs) within the propeller domain of the parasite’s *Pf*kelch13 gene have been associated with the resistant phenotype [6]. These *Pf*kelch13 SNPs were demonstrated to decrease artemisinin sensitivity when inserted into Cambodian isolates [7]. The function/s of the *Pf*kelch13 protein is unclear, though it appears to play a role in triggering the unfolded protein response following protein damage [8]. Sustained alterations of the *Pf*kelch13 protein are not anticipated, unless the changes serve to evade or counteract the mechanisms of action of artemisinin (that is, disrupting haem detoxification in the digestive vacuole, and producing oxidative damage by protein/haem alkylation) [9].

The *Pf*kelch13 coding mutation most strongly associated with artemisinin resistance (C580**Y**) is found throughout South East Asia and is approaching fixation in western Cambodia [6, 10]. Resistance mutations have emerged independently in several South East Asian populations [11]. Nonsynonymous *Pf*kelch13 mutations outside of South East Asia are rare. In Africa the most commonly observed mutation is the A578**S** (reported at 3.8%, KARMA study 2014). This mutation has not been shown to delay parasite clearance and did not confer artemisinin resistance when inserted into a Dd2 parasite line collected from South East Asia [12].

South East Asia has historically acted as a cradle for antimalarial resistance. Resistance to drugs such as chloroquine and sulfadoxine-pyrimethamine initially emerged in South East Asia, then later disseminated to other endemic areas [13]. There is a significant risk that the artemisinin resistant phenotypes within the greater Mekong subregion may similarly spread to other endemic regions.

Molecular surveillance is an important tool for resistance containment. Monitoring clinical outcomes alone may not facilitate the expedient identification and elimination of resistance phenotypes needed to forestall wide-spread resistance. Molecular surveillance is especially vital in sub Saharan Africa, where the majority of disease burden falls, and where immunity is likely to mask treatment failure [14].

Molecular surveillance capacity has yet to be achieved for several endemic regions. Molecular data is typically collected by independent studies, and not ongoing routine surveillance. Here travellers represent a convenient resource to include neglected regions in monitoring of drug resistance [15]. Associated patient data allows genotyping results to be reconciled with epidemiologically important data such as prophylaxis use and specific locations visited (for purposes of reporting novel resistance genotype emergence).

This study aimed to create a profile of epidemiologically relevant information from analysing individual patient data, and to characterise the *Pf*kelch13 propeller domains of *P*. *falciparum* found in whole blood samples (n = 153) archived at the New South Wales (NSW) malaria reference lab from travelers returning with *P*. *falciparum* malaria over the period 2010 to 2016.

## Materials and methods

### Samples

The study used 153 *P*. *falciparum* positive whole blood samples (diagnosed by microscopy), archived 2010 to 2016 at the NSW parasitology reference laboratory at Westmead Hospital, Sydney, NSW. A single blood sample was available for 127 cases, and 13 cases had stored both a blood sample from before and after commencement of treatment. Available patient data had been previously collected by patient interview and recorded within clinician’s notes. Accession numbers were assigned to samples, and epidemiologically relevant decoded patient data were recorded (see Table A in S1 File). As an experimental control, DNA from lab reference strain 3D7 *P*. *falciparum* kindly provided by Dr Jutta Marfurt (Menzies School of Health Research, NT, Australia) was included. Samples were stored at -20°C when not in use. This research was conducted under the ethics approval number LNR/16/WMEAD/62 granted by the Westmead Research Governance Office, Western Sydney Local Health District, NSW, Australia.

### DNA extraction

Genomic DNA was extracted from whole blood samples using a GenElute^™^ Mammalian Genomic DNA Miniprep Kit (Sigma-Aldrich, USA) as per manufacturer’s directions. DNA quality was confirmed by subjecting DNA to gel electrophoresis. DNA concentrations were measured by spectrophotometric analysis using a Nanodrop^®^ Spectrophotometer ND-1000 at 260nm and 280nm.

### *Pf*kelch13

Amplification of the propeller region of the *Pf*kelch13 gene was adapted from Kamau *et al*. 2015 [16]. GeneDB accession number PF3D7_1343700 (http://www.plasmodb.org; accessed 6 July 2016) was used as a reference sequence in these studies, as this curated reference strain is artemisinin susceptible, and contains no *Pf*kelch13 mutations. The primers used were outer primers (Forward: 5′ GCCTTGTTGAAAGAAGCAGAA 3′, Reverse: 5′ CGCCATTTTCTCCTCCTGTA 3′) capturing codon 427–691 of the *Pf*kelch13 propeller gene, semi-nested primers (Forward: 5′ GCCTTGTTGAAAGAAGCAGAA 3′, Reverse: 5′ GTG GCAGCTCCAAAATTCAT 3′) capturing codon 427–676.

The PCR reaction conditions were: Primary Master Mix (MM1) of 25μl total reaction volume containing 2.5μl 10 x PCR buffer (100 mM Tris-HCL, pH 8.3, 500 mM KCl, 15 mM MgCl_2_, 0.01% w/v gelatine), 0.75 μl 50mM MgCl_2_, 2μl 2mM dNTP mix (dinucleotide triphosphates, containing 3 mM dATP and dTTP, 1 mM dCTP and dGTP,), 0.625μl outer forward primer (10μM), 0.625μl outer reverse primer (10μM), 0.4μl BioTAQ DNA polymerase (5U/μl) plus 11.1 μl of sterile water. Template DNA = 7μl/reaction.

The outer PCR product (5μl) was added to Secondary Master Mix (MM2) of the same component concentrations of MM1 except that different reverse primer (semi-nested reverse primer) was used. The reaction volume was made up to 50 μl for the semi-nested PCR.

All reactions were accompanied by a negative control containing sterile water, and otherwise identical reaction conditions.

Amplification was carried out in a Sensoquest Labcycler thermal cycler, with cycling conditions outer round as follows: initial denaturation at 95°C for 15 minutes, followed by 35 cycles of denaturation at 95°C for 1 minute, annealing at 59°C for 1 minute, and extension at 72°C for 2 minutes, with a final extension at 72°C for 10 minutes; semi-nested round: initial denaturation at 95°C for 15 minutes, followed by 40 cycles of denaturation at 95°C for 30 seconds, annealing at 60°C for 1 minute, and extension at 72°C for 1 minutes, with a final extension at 72°C for 10 minutes.

### Geotyping

Primers were designed using Primer3 (http://simgene.com/Primer3) to amplify 15 regions within the mitochondrial and apicoplast *P*. *falciparum* genomes. The 15 amplicons were chosen to capture 23 neutrally inherited SNPs which create a highly predictive geographically informative barcode [17]. Primers and PCR conditions can be found in Table B and Fig A in S1 File. Geotyping amplicon sequences were aligned to *P*. *falciparum* 3D7 reference organellar genome sequences Pf_M76611 (mitochondrial) and PfC10_API_IRAB (apicoplast) (http://www.plasmodb.org; accessed May 2nd, 2017).

### SNP analysis

*Pf*kelch13 and geotyping PCR products were sent to Macrogen (South Korea), for bidirectional sequencing. Sequence data were validated by BLASTN and BLASTX searches (https://blast.ncbi.nlm.nih.gov/Blast.cgi). Sequences were cropped of low quality ends using 4Peaks (http://nucleobytes.com/4peaks/). Trusted regions of reverse sequences were processed using ReverseComplement (http://www.bioinformatics.org/sms/rev_comp.html). All generated sequences per sample were combined to form a sample consensus sequence (contig) using 3cap (http://doua.prabi.fr/software/cap3). Patient contigs were then aligned (using the MEGA7 integrated MUSCLE multiple sequence alignment program) to the reference sequence to identify polymorphisms. *Pf*kelch13 variants were compared to reported resistance-associated mutations [18]. Geotyping SNP locations were compared to distinct haplotypes of the geographically informative barcode (see Fig A in S1 File) [17].

### Accession numbers

**S**equences for the *Pf*kelch13 propeller domain determined from each isolate can be found in GenBank; accession numbers for patients 1–153 are MF076071—MF076223 respectively.

## Results

### Kelch propeller characterization

146 samples aligned identically to the reference *Pf*kelch13 gene ID PF3D7_1343700, including all duplicate samples (collected before and after treatment). Variance from the reference *Pf*kelch13 gene was found in 6 samples originating from Africa (non-coding SNPs) as well as in one sample from Papua New Guinea (coding SNP) as detailed in Table 1.

**Table 1:** Mutations found in *Pfkelch13* propeller domain. SNPs found within *Pfkelch13* propeller consensus sequences (n = 153) as compared to reference 3D7 *P*. *falciparum* sequence.

Isolate	Codon	Nucleotide Change?	Amino Acid Change?	Parasite Origin
BDA57	469	CA**A** -> CA**G**	Glutamine (synonymous)	Nigeria
BDA64	469	CA**A** -> CA**G**	Glutamine (synonymous)	Africa
BDA85	474*	AC**A** -> AC**T**	Threonine (synonymous)	Ghana (Dakwa)
BDB3	477	TC**T** -> TC**G**	Serine (synonymous)	Zambia
BDA83	491	TT**C** -> TT**T**	Phenylalanine (synonymous)	West Kenya
BDB5	580**	T**G**T -> T**A**T	Cysteine -> Tyrosine	Papua New Guinea
BDA60	621	GC**T** -> GC**A**	Alanine (Synonymous)	Nigeria

Notes:

*The non-synonymous T474**I** SNP at this codon site has previously been associated with artemisinin resistance [7].

** The C580**Y** kelch mutation is the molecular marker most strongly associated with artemisinin resistance [8].

### Resistance-associated genotype

The protein translations of variant sequences were compared to the known resistance-associated *Pf*kelch13 alleles. This revealed the nucleotide polymorphism found in isolate BDB5, extracted from a traveler returning from Papua New Guinea, resulted in the C580**Y** artemisinin resistance mutation.

### Geotyping

**Fig 1:**
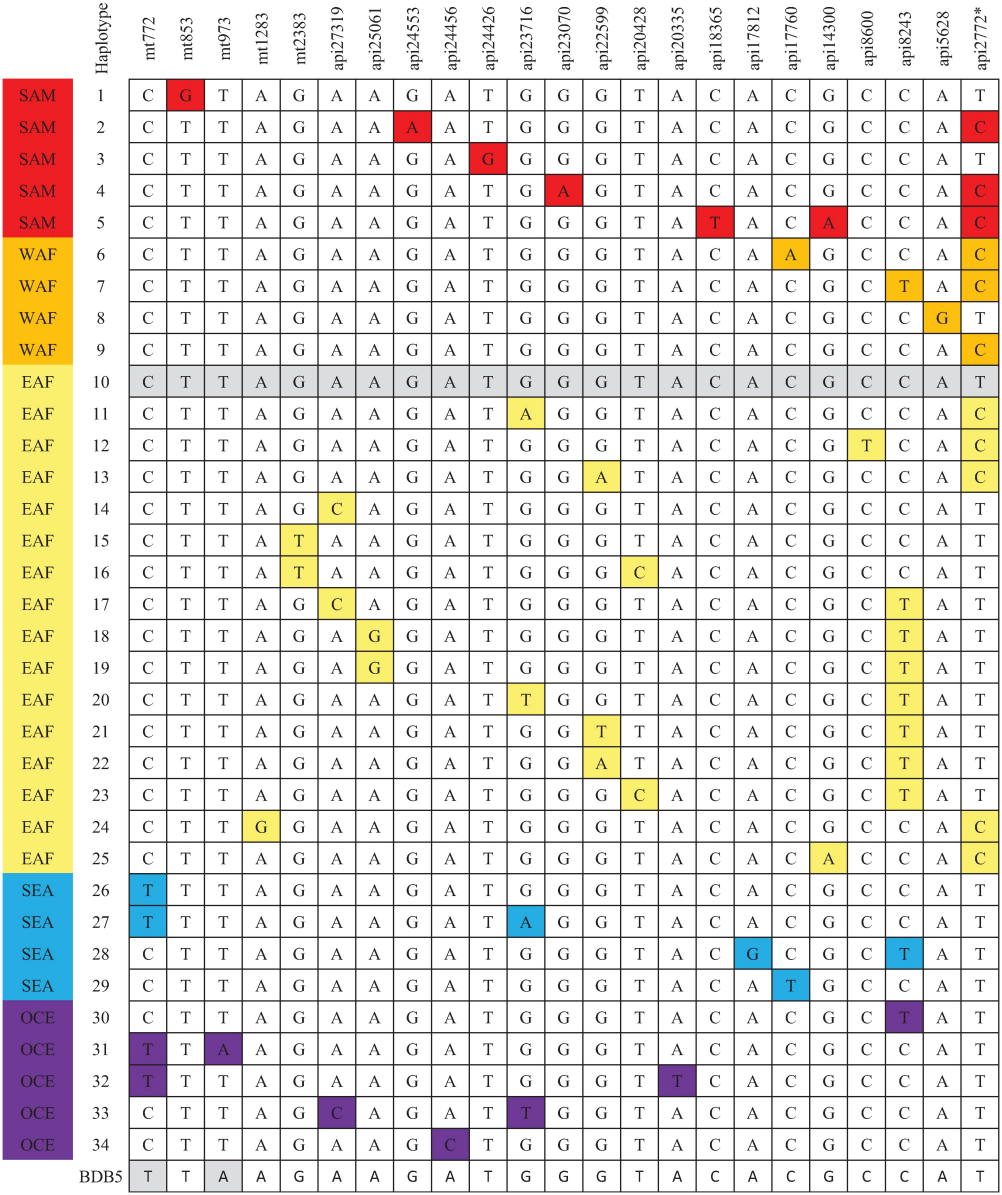
Geographically informative barcode of *P*. *falciparum* organellar genome SNPs. The C580**Y** Papua New Guinea (BDB5) sample sequence results for the 23 predictive geotyping SNP *loci* are compared to the geographically informative haplotype barcodes previously defined (predictive accuracy 92.1%) [17]. SAM (South America); WAF: West Africa; EAF: East Africa; SEA: South East Asia, OCE: Oceania. Haplotype 10 represents the 3D7 reference *P*. *falciparum*. *SNP *loci* api2772 additionally occurs at nucleotide position api31461due to an inverted repeat within the apicoplast genome.

The barcode generated for the mutant Papua New Guinea sample (isolate BDB5) aligned to a haplotype unique to Oceania, as shown below in Fig 1.

### Patient profile

Most specimens originated from Africa (n = 141). A considerable proportion of the African cohort travelled to Australia from Nigeria (n = 28) or Sudan (n = 26). Additional African countries included Kenya (n = 15), Ghana (n = 13), Sierra Leone (n = 5), and the Gambia (n = 5). African countries of origin were grouped largely to the west and east geographical regions of the continent. Travellers included refugees as well as immigrants having visited rural areas to meet family obligations (Table A in S1 File).

Six patients had travelled to Papua New Guinea, two patients from both Indonesia and India, one patient returned from Peru, and one patient returned from Thailand.

The study population was composed of 121 males and 32 females, with a mean age of 37.54±10, ranging from 6 months to 79 years old (at time of infection). The mean parasitaemia was 1.55% erythrocytes infected, with less than 24% of samples <0.01%. Few patients (where reported) had used chemoprophylaxis (doxycycline, artemether-lumefantrine, or mefloquine). Instances of co-morbidity were low (reported in less than 5% of cases, most commonly dengue fever).

## Discussion

Considering the significant health threat artemisinin resistance represents, there is a consensus to upscale surveillance of molecular markers outside of South East Asia [19]. The *Pf*kelch13 molecular surveillance studies which have been conducted in Africa thus far do not include Zimbabwe or Sudan (origins of parasites examined in this study) [20]. Recently, ongoing resistance surveillance has begun in 15 African locations (reporting the Plasmodium Diversity Network of Africa) [18]; in this study, parasites were screened from origins that are not yet included in this network (Nigeria, Sudan, Sierra Leone, Zambia, Malawi, Uganda, Mozambique, Zimbabwe, Benin, and Togo).

The age and sex distribution of the cohort differed from that of studies conducted within endemic regions, as is expected with a large proportion of non-immune travelers [21]. The substantial gender bias observed in this study is unlikely to denote a controverting factor, given men and women historically present differing travel-related morbidity profiles [22]. The current Australian treatment guidelines for uncomplicated *P*. *falciparum* malaria recommend Artemether-lumefantrine (Riamet^®^, Coartem^®^) as the frontline treatment, or as a second choice Atovaquone-proguanil (Malarone^®^) (in cases where atovaquone-proguanil has not been taken as prophylaxis), and as a last choice alternative Quinine with clindamycin. All patients within this study, including the patient from whom the C580**Y** sample (BDB5) was collected, responded to treatment with no evidence of delayed parasite clearance.

The low level of *Pf*kelch13 propeller domain nucleotide diversity found in this study conforms to expectations [23]. No coding mutations were observed in African parasites, which is consistent with most *Pf*kelch13 investigations of natural *P*. *falciparum* populations [16, 18, 24]. This is expected in the absence of widespread artemisinin use, as there is presumably a purifying selection for the unaltered allele in the absence of a selective drug pressure [25].

The C580**Y** isolate (BDB5) originated from Papua New Guinea, where there is no report of delayed parasite clearance with artemisinins, and there has been no previous observation of this or any other resistance-associated *Pf*kelch13 mutations to our knowledge. Resistance genotypes have additionally not been reported in countries adjacent to both Papua New Guinea and the greater Mekong subregion, where resistant parasites would likely be observed before Papua New Guinea in the instance of typical dissemination [26]. Clinical investigation by the Tracking Resistance to Artemisinins Collaboration (TRAC) and molecular investigation conducted by the MalariaGEN *Plasmodium falciparum* project confides the perimeter of artemisinin resistance to the greater Mekong subregion [16, 4].

There is a historical precedence both for native Papua New Guinea *P*. *falciparum* to give rise to *de novo* resistance, as well as to acquire resistance from South East Asia via human migration and the three anopheles vector species common to both regions [27]. Resistance to pyrimethamine and chloroquine both emerged within Papua New Guinea independently, and individually migrated from South East Asian locations [28].

The geotyping result identified the Papua New Guinea sample as haplotype #31 (Fig 1), a haplotype unique to Oceania [17]. This result predicts that the sample is indigenous to Oceania, rather than having evolutionary origins within the artemisinin resistance perimeter. It is possible the indigenous Papua New Guinea parasite generated the coding mutation *de novo*, or potentially recombined with a migrating lineage from the greater Mekong subregion. The patient did not experience treatment failure with ACT. It cannot be assumed that the C580**Y** *Pf*kelch13 mutation alone conferred resistance in this case. The capacity for natural *P*. *falciparum* populations, including those of the pacific region, to generate artemisinin resistance is unclear. Additional molecular markers may be necessary to discern artemisinin resistance outside South East Asia [29].

Resistance to conventional antimalarial drugs has previously spread though Papua New Guinea in a hard selective sweep; that is, a single allele spreading through a population as opposed to multiple adaptive alleles [30]. Genome Wide Association Studies conducted in 2015 found four background alleles (ferredoxin, apicoplast ribosomal protein S10, multidrug resistance protein 2, and chloroquine resistance transporter) strongly associated with kelch-mediated artemisinin resistance. These alleles are consistently present within founder populations in South East Asia [31]. These may be markers of a permissive genome more likely to generate resistance, elements of a multi-loci gene network that modulates susceptibility, or simply artifacts resulting from the recurrent bottlenecking of South East Asian parasites [29, 32]. If artemisinin resistance is selected in a soft sweep, the vast reservoir of variation in natural *P*. *falciparum* populations represents a significant risk of emergent resistance disseminating rapidly. This advocates broader molecular surveillance of *Pfkelch13*, and other relevant markers as they emerge [33]. Screening infected travelers returning from malaria endemic regions represents an opportunity to participate in defining changing genotypes for regions outside South East Asia.

Epidemiologically important data such as chemoprophylaxis use is important to consider, as this may confound the parasite population captured by surveillance. Speculation here is limited, as in many cases use or compliance to prophylaxis was not recorded, as shown in Table A in S1 File. The patient interview format means information such as prophylaxis use is self-reported. A significant limitation is that relevant patient information (including specific location/s visited, previous exposure to malaria) is typically not recorded, with 81% of samples including no additional data outside age, sex, and origin (Table A in S1 File). This could be overcome to some degree by implementing a rudimentary questionnaire for imported malaria cases. This would collect valuable clinical data to enrich genotyping results, while placing nominal burden on healthcare workers.

## Conclusions

Locally, this study identifies a risk of imported resistant *falciparum* malaria in Australia. As resistance to artemisinin and partner drugs continues to increase and spread globally, this risk will increase, and Australia needs to be prepared to respond to this. Ongoing resistance screening will improve case management and contribute to global data gathering efforts.

## Supporting information

S1 File Supplemental materials. Table A. listing genotyping results and patient data, Table B. describing PCR conditions for *P*. *falciparum* barcode geotyping, and Figure A. detailing geotyping amplicon sequences including primers and geotyping SNP loci within *P*. *falciparum* 3D7 and apicoplast reference sequences.
(PDF)
