# Supplementary material for: Resistance screening and trend analysis of imported falciparum malaria in NSW, Australia (2010 to 2016)
Source: PLoS One. 2018 May 29;13(5):e0197369. doi: 10.1371/journal.pone.0197369 (PMC5973583; doi:10.1371/journal.pone.0197369)
Supplement: S1 File — Table A. listing genotyping results and patient data, Table B. describing PCR conditions for P. falciparum barcode geotyping, and Figure A. detailing geotyping amplicon sequences including primers and geotyping SNP loci within P. falciparum 3D7 and apicoplast reference sequences. (PDF) [file pone.0197369.s001.pdf]

Table A. Sample results and patient data.

Patient Sample	Sex	Age	Collection date	GenBank Acc. No.	Isolate[1]	*pfK13*[2]	Country of travel	Parasitaemia (%RBC)	Other[3]
1	F	41	26.02.16	MF076071	BDA1	wt	Zimbabwae, Mosambique	0.16	
2	M	20	17.02.16	MF076072	BDA2	wt	Sudan	0.79	P, TF
3	M	21	20.01.16	MF076073	BDA3	wt	Sudan	0.3	P
4	M	33	16.01.16	MF076074	BDA4	wt	Sudan	0.02	
5	F	49	17.01.16	MF076075	BDA5	wt	Ghana	<0.01	P
**6**	**F**	**13**	**21.04.16**	**MF076076**	**BDA6**	**wt**	**Malawi**	**1.5**	
7	F	48	21.04.16	MF076077	BDA7	wt	Madagascar	30.1	PNC
**8**	**F**	**13**	**11.04.16**	**MF076078**	**BDA8**	**wt**	**Malawi**	**<0.01**	
**9**	**M**	**6**	**~**	**MF076079**	**BDA9**	**wt**	**Malawi**	**0.04**	
**10**	**F**	**6**	**~**	**MF076080**	**BDA10**	**wt**	**Malawi**	**1.5**	
11	M	43	17.12.15	MF076081	BDA11	wt	Nigeria	0.05	
**12**	**M**	**58**	**24.11.15**	**MF076082**	**BDA12**	**wt**	**Cameroon**	**4.13**	**P**
**13**	**M**	**58**	**24.11.15**	**MF076083**	**BDA13**	**wt**	**Cameroon**	**3.2**	**P**
14	M	35	16.11.15	MF076084	BDA14	wt	Mali	~	
15	M	38	03.11.15	MF076085	BDA15	wt	Nigeria	0.2	
16	M	32	03.11.15	MF076086	BDA16	wt	Sudan	1.7	P
17	M	47	22.10.15	MF076087	BDA17	wt	Nigeria	0.15	
18	M	38	18.10.15	MF076088	BDA18	wt	Mali	1.29	
19	F	40	04.09.15	MF076089	BDA19	wt	Sudan	0.2	P
20	M	34	14.08.15	MF076090	BDA20	wt	Thailand, Laos	0.19	
21	M	37	13.08.15	MF076091	BDA21	wt	Sudan	<0.01	
22	M	37	13.08.15	MF076092	BDA22	wt	Sudan	0.2	
23	M	37	13.08.15	MF076093	BDA23	wt	Sudan	1.2	
24	M	26	10.08.15	MF076094	BDA24	wt	Nigeria	1.1	
25	M	26	09.08.15	MF076095	BDA25	wt	Nigeria	0.36	
26	F	37	14.07.15	MF076096	BDA26	wt	Sudan	<0.01	
27	M	44	03.06.15	MF076097	BDA27	wt	Sudan	0.02	PNC
28	M	47	24.03.15	MF076098	BDA28	wt	Tanzania	~	CM
29	M	49	13.04.15	MF076099	BDA29	wt	Nigeria	1.1	
30	M	57	17.03.15	MF076100	BDA30	wt	PNG	<0.01	P, TF
31	M	57	21.02.15	MF076101	BDA31	wt	Zambia	0.2	
32	M	26	07.02.15	MF076102	BDA32	wt	PNG	0.3	CM
33	F	8	06.02.15	MF076103	BDA33	wt	Sudan	1.29	
34	F	8	06.02.15	MF076104	BDA34	wt	Sudan	0.6	
35	F	29	31.01.15	MF076105	BDA35	wt	PNG	0.3	
36	M	51	19.06.14	MF076106	BDA36	wt	Sumatra	1.7	
37	M	70	18.09.14	MF076107	BDA37	wt	Sudan	<0.01	
38	M	42	13.12.14	MF076108	BDA38	wt	Cameroon	<0.01	
39	F	68	27.12.14	MF076109	BDA39	wt	Sudan	~	TF
40	F	57	27.09.14	MF076110	BDA40	wt	South Africa	~	P
41	M	44	17.09.14	MF076111	BDA41	wt	Sudan	0.06	
**42**	**M**	**41**	**25.08.14**	**MF076112**	**BDA42**	**wt**	**Kenya**	**0.12**	**PNC**
43	F	23	13.08.14	MF076113	BDA43	wt	Sierra Leone	12.25	
44	M	37	16.07.14	MF076114	BDA44	wt	Nigeria	1.01	
45	F	13	01.07.14	MF076115	BDA45	wt	Ghana	0.14	
46	F	41	01.07.14	MF076116	BDA46	wt	Ghana	0.64	PNC
47	M	43	30.06.14	MF076117	BDA47	wt	Tanzania	<0.01	
48	F	33	20.06.14	MF076118	BDA48	wt	Nigeria	<0.01	
**49**	**M**	**42**	**01.06.14**	**MF076119**	**BDA49**	**wt**	**Sierra Leone**	**<0.01**	
50	M	31	19.05.14	MF076120	BDA50	wt	Togo, Benin	0.27	

51	F	38	19.05.14	MF076121	BDA51	wt	Sudan	0.67	
52	F	28	12.05.14	MF076122	BDA52	wt	Peru	0.4	CM
53	M	35	09.05.14	MF076123	BDA53	wt	Sudan	0.09	
54	M	30	27.04.14	MF076124	BDA54	wt	Uganda	1.07	
55	M	28	27.03.14	MF076125	BDA55	wt	Africa	~	
56	M	22	03.03.14	MF076126	BDA56	wt	Sierra Leone	0.1	
57	M	44	15.03.14	MF076127	BDA57	469 CA**A**->CA**G**	Africa	1.4	
**58**	**F**	**27**	**29.01.14**	**MF076128**	**BDA58**	**wt**	**Kenya**	**0.1**	**CM**
59	M	55	22.01.14	MF076129	BDA59	wt	West Kenya	1	PNC
60	M	37	20.01.14	MF076130	BDA60	621 GC**T**->GC**A**	Nigeria	0.61	P
61	M	28	07.01.14	MF076131	BDA61	wt	Kenya	1.4	
62	M	28	08.01.14	MF076132	BDA62	wt	Kenya	<0.01	
63	M	28	09.01.14	MF076133	BDA63	wt	Kenya	<0.01	
64	M	25	14.02.14	MF076134	BDA64	469 CA**A**->CA**G**	Africa	<0.01	
**65**	**M**	**57**	**28.12.13**	**MF076135**	**BDA65**	**wt**	**Nigeria, China**	**1**	
66	M	52	26.11.13	MF076136	BDA66	wt	Africa	1	
67	M	30	21.11.13	MF076137	BDA67	wt	Ghana	0.12	PNC
**68**	**M**	**20**	**05.08.13**	**MF076138**	**BDA68**	**wt**	**South Sudan**	**0.4**	
**69**	**M**	**20**	**05.08.13**	**MF076139**	**BDA69**	**wt**	**South Sudan**	**1.5**	
70	M	38	23.07.13	MF076140	BDA70	wt	Ghana	1.1	CM
71	M	13	20.07.13	MF076141	BDA71	wt	Kenya	0.4	
72	M	19	19.07.13	MF076142	BDA72	wt	Kenya	<0.01	
73	M	28	16.07.13	MF076143	BDA73	wt	Kenya	0.4	
**74**	**M**	**1**	**18.06.13**	**MF076144**	**BDA74**	**wt**	**Kenya**	**7.6**	
75	F	44	06.06.13	MF076145	BDA75	wt	Ivory Coast	<0.01	
76	F	44	06.06.13	MF076146	BDA76	wt	Ivory Coast	0.17	
77	M	31	22.05.13	MF076147	BDA77	wt	Nigeria	<0.01	
78	M	37	19.04.13	MF076148	BDA78	wt	Nigeria	~	
79	M	37	20.04.13	MF076149	BDA79	wt	Nigeria	1	
80	M	37	20.04.13	MF076150	BDA80	wt	Nigeria	<0.01	
81	M	65	21.03.13	MF076151	BDA81	wt	Africa	0.9	
82	M	28	17.02.13	MF076152	BDA82	wt	Tanzania	0.9	PNC
**83**	**M**	**39**	**12.01.13**	**MF076153**	**BDA83**	**491** TTC->TTT	**West Kenya**	**<0.01**	**PNC**
84	F	47	08.01.13	MF076154	BDA84	wt	Sierra Leone	0.2	
**85**	**M**	**45**	**11.12.12**	**MF076155**	**BDA85**	**474** ACA->ACT	**Ghana, Dakwa**	**3.7**	**PNC**
86	M	44	19.12.12	MF076156	BDA86	wt	Nigeria	<0.01	P
87	M	40	26.10.12	MF076157	BDA87	wt	Nigeria	1.1	
88	M	34	20.10.12	MF076158	BDA88	wt	Africa	0.9	
89	M	38	03.10.12	MF076159	BDA89	wt	Africa	<0.01	
90	F	67	10.10.12	MF076160	BDA90	wt	Africa	1.2	
91	M	31	12.10.12	MF076161	BDA91	wt	Nigeria	2.5	
92	M	33	11.10.12	MF076162	BDA92	wt	Nigeria	4	
93	M	33	14.10.12	MF076163	BDA93	wt	Nigeria	0.1	
94	M	24	06.07.12	MF076164	BDA94	wt	Nigeria	<0.01	
95	M	55	14.08.12	MF076165	BDA95	wt	Sudan	3	
96	M	57	20.07.12	MF076166	BDA96	wt	Ghana	1.1	
**97**	**F**	**67**	**01.09.12**	**MF076167**	**BDA97**	**wt**	**South Sudan**	**0.03**	
98	M	57	18.07.12	MF076168	BDA98	wt	Ghana	1	
99	M	48	06.07.12	MF076169	BDA99	wt	Africa	0.46	
100	M	38	04.10.12	MF076170	BDA100	wt	Africa	<0.01	
101	M	57	08.04.12	MF076171	BDB1	wt	Africa	2	
102	M	54	23.03.12	MF076172	BDB2	wt	Africa	11	
103	M	52	23.04.12	MF076173	BDB3	477 TC**T**->TC**G**	Zambia	<0.01	
104	M	48	07.07.12	MF076174	BDB4	wt	Africa	~	

105	M	16	14.02.12	MF076175	BDB5	580 TGT->TAT	PNG	<0.01	
106	M	45	28.01.12	MF076176	BDB6	wt	PNG	2.81	
107	M	20	21.01.12	MF076177	BDB7	wt	Nigeria	<0.01	
108	M	20	21.01.12	MF076178	BDB8	wt	Nigeria	<0.01	
109	M	20	22.01.12	MF076179	BDB9	wt	Africa	1.1	
110	M	35	05.01.12	MF076180	BDB10	wt	Africa	<0.01	
111	F	63	18.12.11	MF076181	BDB11	wt	Africa	<0.01	
112	M	38	09.12.11	MF076182	BDB12	wt	PNG	0.7	
113	~	17	~	MF076183	BDB13	wt	Sierra Leone	0.06	PNC
**114**	**M**	**34**	**08.11.11**	**MF076184**	**BDB14**	**wt**	**Sudan**	**0.5**	**PNC**
115	M	5	01.11.11	MF076185	BDB15	wt	Sudan	0.8	
116	M	24	31.10.11	MF076186	BDB16	wt	Nigeria	0.2	
117	M	7	07.10.11	MF076187	BDB17	wt	Africa	~	
118	M	74	04.10.11	MF076188	BDB18	wt	Kenya	2.3	
119	~	42	~	MF076189	BDB19	wt	Kenya	-	
120	F	30	08.09.11	MF076190	BDB20	wt	Africa	2.4	
121	~	42	~	MF076191	BDB21	wt	Kenya	~	
122	M	79	02.09.11	MF076192	BDB22	wt	Nigeria	<0.01	
123	M	79	01.09.11	MF076193	BDB23	wt	Nigeria	0.3	
124	M	39	12.08.11	MF076194	BDB24	wt	Ghana	<0.01	
**125**	**M**	**72**	**22.07.11**	**MF076195**	**BDB25**	**wt**	**Nigeria**	**~**	**TF**
126	M	45	25.07.11	MF076196	BDB26	wt	Ghana	4.27	
127	~	56	~	MF076197	BDB27	wt	Sudan	0.14	
128	M	36	26.06.11	MF076198	BDB28	wt	Sudan	2	P
129	F	44	30.04.11	MF076199	BDB29	wt	Ghana	0.1	P
130	F	34	19.05.11	MF076200	BDB30	wt	Kenya	0.27	
131	M	29	15.03.11	MF076201	BDB31	wt	Nigeria	0.08	CM
132	F	55	09.04.11	MF076202	BDB32	wt	Zambia	0.04	
133	F	19	~	MF076203	BDB33	wt	Gambia	3.4	
134	~	20	~	MF076204	BDB34	wt	PNG	0.8	
135	M	52	08.02.11	MF076205	BDB35	wt	Africa	<0.01	
136	F	19	10.01.11	MF076206	BDB36	wt	Gambia	1.9	
137	M	31	15.12.10	MF076207	BDB37	wt	Ghana	<0.01	
138	M	4	16.09.10	MF076208	BDB38	wt	Africa	0.3	
139	~	10	~	MF076209	BDB39	wt	Gambia	0.15	
140	~	61	~	MF076210	BDB40	wt	Gambia	<0.01	
141	~	0	~	MF076211	BDB41	wt	Gambia	0.03	
142	F	20	20.10.10	MF076212	BDB42	wt	Africa	<0.01	
143	M	52	17.10.10	MF076213	BDB43	wt	Indonesia (Lombek)	2	
**144**	**M**	**40**	**12.08.10**	**MF076214**	**BDB44**	**wt**	**Nigeria**	**<0.01**	
145	M	45	15.07.10	MF076215	BDB45	wt	Africa	0.04	
146	M	30	13.03.10	MF076216	BDB46	wt	Africa	0.01	
147	M	30	03.04.10	MF076217	BDB47	wt	Uganda	1.2	
148	M	70	28.07.10	MF076218	BDB48	wt	India	<0.01	
149	M	70	28.07.10	MF076219	BDB49	wt	India	0.1	
150	M	36	02.06.10	MF076220	BDB50	wt	Sudan	0.63	
151	F	49	01.06.16	MF076221	BDB51	wt	Ghana	<0.01	P
152	F	49	04.06.16	MF076222	BDB52	wt	Ghana	6.7	
**153**	**M**	**41**	**15.06.16**	**MF076223**	**BDB53**	**wt**	**Sudan**	**3.7**	**PNC**

Notes:[1]Isolate ID number for biological materials collection; [2]*pfK13* sequencing result, wt = wild type allele, sequences that differ from *P. falciparum* 3D7 reference shown by codon number for site of mutation, and nucleotide change (wt->mutation); [3]Additional metadata: P = prophylaxis compliance, PNC = prophylaxis non-compliance, CM = reported co-morbidity, TF = previous treatment failure.

Boxed patients = kelch propeller domain contained SNP.

Bold patients = patient is known to be a refugee or immigrant native to country of parasite origin.

Table B. PCR conditions for *P. falciparum* barcode geotyping. SNP *loci* targets within the mitochondrial and apicoplast *P. falciparum* genomes amplified for geographically informative barcoding (mitochondria reference sequence: Pf_M76611 (5967bp), apicoplast reference sequence PfC10_API_IRAB (34242bp), plasmodb.org accessed Dec 1st, 2017). PCR conditions are listed where they vary from the following reaction conditions: all reactions were made up to 50µl total reaction volume, including template DNA = 6µl/reaction, and components were added to make a final concentration per reaction of: 1X conc. PCR buffer (100 mM Tris-HCL, pH 8.3, 500 mM KCl, 15 mM MgCl2, 0.01 % w/v gelatine), 2mM dNTP mix, 0.25µM forward and reverse primers, and 2U BioTAQ DNA polymerase. Amplification was carried out in a Sensoquest Labcycler thermal cycler, with programmes including initial denaturation at 95°C for 15 minutes, cycle conditions as given below, and a final extension at 72°C for 15 minutes.

Barcode target	Amplicon	Primers	$MgCl_2$	T_d	T_a	T_e	Cycles
mt772, mt853, mt973, mt1283	Pf_MtA<br>n: 611–1542	F: 5′ TTCCCTTCTCGCCATTTGAT 3′<br>R: 5′ GGCGAACCTTCTTACCGTTAT 3′	1.5 mM	94ºC<br>30s	50ºC<br>30s	72ºC<br>60s	30<br>X
mt2383	Pf_MtB<br>n: 1929–2432	F: 5′ TTAACGCCTGACATGGATGG 3′<br>R: 5′ CATCCAGTTCCACCACCAAA 3′	1.5 mM	94ºC<br>30s	50ºC<br>30s	72ºC<br>60s	30<br>X
api27319	Pf_ApiA<br>n: 27135-28042	F: 5′ GGTCCAAAATTACAAGCACCCT 3′<br>R: 5′ GGTCGAAAGCAATGGACTCA 3′	1.5 mM	94ºC<br>30s	59ºC<br>30s	72ºC<br>60s	30<br>X
api25061	Pf_ApiB<br>n: 24661-25588	F: 5′ AGATCCTTTTCCTGATCCCATT 3′<br>R: 5′ GATATGGGACAAAAAGTACATCCT 3′	2.0 mM	94ºC<br>30s	54.5ºC<br>30s	72ºC<br>60s	30<br>X
api24426, api24456, api24553	Pf_ApiC<br>n: 24167-24681	F: 5′ AACTACTCCTATAACTATATCACCA 3′<br>R: 5′ TGGGATCAGGAAAAGGATCT 3′	2.0 mM	94ºC<br>30s	45ºC<br>30s	72ºC<br>60s	30<br>X
api23716	Pf_ApiD<br>n: 22668-24022	F: 5′ TCTAATTCTTCCTTTTTTTATTGT 3′<br>R: 5′ AATTTTGTAGGTACTAAAAGTAAT 3′	2.0 mM	94ºC<br>60s	40ºC<br>60s	72ºC<br>120s	35<br>X
api23070	Pf_ApiE<br>n: 22614-23593	F: 5′ TCCTAATCCTATCCAACCTTGT 3′<br>R: 5′ AGCTTTAAAATATAAAATTGGTGGT 3′	2.0 mM	94ºC<br>60s	56ºC<br>60s	72ºC<br>120s	35<br>X
api22599	Pf_ApiF<br>n: 21942-22636	F: 5′ ATCATTGTGTTTGCTTATGTTTTTT 3′<br>R: 5′ AACAAGGTTGGATAGGATTAGG 3′	1.5 mM	94ºC<br>30s	52ºC<br>30s	72ºC<br>60s	30<br>X
api20428, api20335	Pf_ApiG<br>n: 19673-20625	F: 5′ AAAGGTTTATGACGACCACCT 3′<br>R: 5′ ATAGGGCATGTAGATCATGGAA 3′	1.5 mM	94ºC<br>30s	50ºC<br>30s	72ºC<br>60s	30<br>X
api18365	Pf_ApiH<br>n: 17895-18917	F: 5′ ATTATCATTTACCCCTTCTAGTATT 3′<br>R: 5′ GGTTCGAATCCTTTAGGACA 3′	1.5 mM	94ºC<br>60s	42.5ºC<br>60s	72ºC<br>120s	35<br>X
api17812, api17760	Pf_ApiI<br>n: 16998-17929	F: 5′ ACCACTAGGCCCACATAAAA 3′<br>R: 5′ AAAAACATATAATACTAGAAGGGGT 3′	1.5 mM	94ºC<br>60s	55ºC<br>60s	72ºC<br>120s	35<br>X
api14300	Pf_ApiJ<br>n: 14267-14705	F: 5′ ATTTATCAAGCAACTAAAAGTTCA 3′<br>R: 5′ ACCATCCATTACCAACAGGT 3′	2.0 mM	94ºC<br>60s	56ºC<br>60s	72ºC<br>120s	35<br>X
api8600, api8243	Pf_ApiK<br>n: 8159-8771	F: 5′ GAAGGATTAACTTGTGGTTTAGTT 3′<br>R: 5′ TAACTTTTTCCCCTACCCATACA 3′	2.0 mM	94ºC<br>30s	45ºC<br>30s	72ºC<br>60s	30<br>X
api5628	Pf_ApiL<br>n: 5095-6038	F: 5′ AGTTCAAATCTCACCATTAGCTT 3′<br>R: 5′ AATCGAACCTACTTCAACTTGT 3′	1.5 mM	94ºC<br>30s	54.5ºC<br>30s	72ºC<br>60s	30<br>X
api2772	Pf_ApiM<br>n: 2271-3084	F: 5′ GGTTCAATTCCGTTTTTCGC 3′<br>R: 5′ AATTCGATTGGCATTTCACCA 3′	1.5 mM	94ºC<br>30s	54.5ºC<br>30s	72ºC<br>60s	30<br>X

Figure A. Geotyping amplicon sequences for Plasmodium falciparum 3D7, mitochondria reference sequence: Pf_M76611 (5967bp), apicoplast reference sequence PfC10_API_IRAB (34242bp) (plasmodb.org accessed Dec 1st, 2017). Primers, Geotyping SNP loci.

```
>Pf_MtA | Plasmodium falciparum 3D7 | 932bp (n: 611 - 1542)
      Features: Geotyping sites mt772, mt853, mt973, & mt1283

TTCCCTTCTCGCCATTTGATAGCGGTTAACCTTTCCTTTTCCTTACGTACTCTAGCTATGAACACAATTGTCTAT
TCGTACAATTATTCATATATATATTTGAAACAGGACATACATGTTCATTTATTCTGAATAGAATAAGAACTCTAT
AAATAACCAGACTATTTCAACAAAATGCCAATATAAAATTGTAATTTGATCAGTGTGAGGTATAACAATATATGA
TATACCGAAAGAATTTATAAACCATTCGGTAGAAGTATCATATATTTCTATTATTCTTATAAAGTATATTATTAA
TAATAATAAACCTATTACTACATGAGAAAAATGTAATCCTGTAACACAATAAAATAATGTAGTATATACAGTATC
ATTTATATGATATGATAAATGTAAATACTCTGTAGTTTGTAGAGATGCAAAACATTCTCCTAATAAGTATATTAT
ACAAATAATACTAGAGATTTCAAAACTCATTCCTTTTTCTATAAATACTTGTAAACATGCAGTCATACATGATGC
ACTAGCTAATATAAATGTAATTGTTAAGATTAACATTCTTGATGAAGTAATGATAATACCTTCATTACTTAATGG
ATATGGTGATAAACTAAAATGTAATATACCCCAAAAATATGTAAAGAATAATAAAGCTTCTGATATTATGATAGA
TAACATACCAGAAGTTAAAGATGAAAATACAGAATAAAAACTTTCTCGAATAGAATATACAAATATTAATAGGAT
TATAGGGTTAAATGTAAATAATATCCCTACAGAAAAGTATTTTAAAGATGTACCATATAATGATGTTAATGCAGG
ATATGAAACTAGATGTGCTTTTATATTTGATAAATTACTAAATAAAATAAATTTATAAGAACGGTGAGATAATGT
GCCGTAAACATATAACGGTAAGAAGGTTCGCC


>Pf_MtB | Plasmodium falciparum 3D7 | 504bp (n: 1929 - 2432)
      Features: Geotyping site mt2383

TTAACGCCTGACATGGATGGATAATACTCGACTCTTCCAAAGTATAACCGCTGTCGCTGGGACTGTATGGATCAA
ATATTTCTCATTTATATCCGAGCCTCATGTTATTTTTATTGTTTTAAATAGATATTCACTTATTACAAATTGTAA
CCATAAAACTTTAGGATTATACTATTTATGGTTTTCATTTTTATTTGGTAGTTATGGATTTTTATTATCAGTAAT
ACTACGTACTGAATTATATTCTTCATCTTTAAGAATAATTGCACAAGAAAATGTAAATCTATATAATATGATATT
TACAATTCACGGAATAATTATGATTTTTTTCAATATAATGCCAGGATTATTCGGAGGATTTGGTAATTACTTTCT
ACCTATTTTATGTGGATCTCCAGAATTAGCATATCCTAGAATTAATAGTATATCTTTACTGTTACAACCAATTGC
TTTTGTTTTAGTTATATTATCTACTGCAGCAGAATTTGGTGGTGGAACTGGATG


>Pf_ApiA | Plasmodium falciparum 3D7 | 908bp (n: 27135 - 28042)
      Features: Geotyping site api27319

GGTCCAAAATTACAAGCACCCTGTTTACATACAGGTGATTTAAAATTTTTTAATCTAGCTTTTCCTAGCCCTTTT
TGTACTCTTATTTTTTTATTACTAAAATATACTTTACTTTTATTCTTAGTATGTTTATACATATAAATATTATAT
ATATATAAATATTTTATAATATAAATTATTAATTTACATATTTTAATATAATTATTAAAATATAATTTAATAAAA
AAATTATATTTATATTTAAATATTATATTATTTAATGTATTATTATTTAAAATAATAATATTCATTCTAAAATAT
ACATATAATATTATTTTCAGAAAAATAGGATTTGAACCTATATTCTTCTATTCCCAAAATAGATATGTTACCATT
ACACTATATTCTGAATTAAATAAATACTTTTAAGGAAAATCGAATTCCTATTTTCTTCTTGAAAAAAAGATGTCT
TACCTTTAAACGATAAAAGTTATTAAATGAACTACCTGAGATTTGAACTCAGAACCATTCGATTAAAAGTCGAGT
ACTCTACCAATTAAGCTAGTAATTCTTAAATAAAACGAATTTGACGAGAATTGAACTCGTATTCTTTATTATGAC
AAAATAATATTTTTACCTAATTAAACTAACAAATTCTAATAATAGAGAAAAAGGGATTCGAACCCTTGGTATAAT
ATTATTATATATACAATAAATTAGCAATTTATAACTATAAACCTCTCAGTCATTTCTCTTTATATATAAAAATAA
GTTAAATCAGATTTGAACTGATGTAGATATATTATCAATGGATTTACAGTCCATTCCTTTTAACCACTCAGGCAT
TAACTTTAAATTACATTTAAGTAGATTCGAACTACTGATGATCAATATATTGAAAATGAATTATGAGTCCATTGC
TTTCGACC
```

>Pf_ApiB | Plasmodium falciparum 3D7 | 927bp (n: 24661 - 25588)
Features: Geotyping site api25061

AGATCCTTTTCCTGATCCCATTCTAGTTTTTAAAGATTTTTTAGTTAATGATTTTATACATCTTATACAAATTTT
ATATACTCCTATTTTTTTTAAATATTTATTTATTATAAATTTTGAAGTCTCTAATTGATTTTTTGTTAAAAATCC
AGAATCTAAAGATATAATACCCCAATATAAACTTAAAAATTTTAAATTAAATTTACCTTTTATTTTACCTTTTTG
ATTTTTTTTTATTATAATATTAGTCATATATTATAAATTTTTTTAATTTATTTTTAAATATTTAACCAAATTTTT
ATACTTAAAATACCATATTTAGTATTTATAATATCATTTATATATTTAATATTATTATTTAATGTACTTAAAGAT
ATAATACCATAAATATATATTTCTATTTTAGTCTTTAAACTATTTTTAAAACGACCTGAAAATTTAATTTTTAAT
CCTTTTATATTAAAATTATTTTTTTTTAAAATATTATTATATAAATAATCAAATATTAATTTTAAAGATTTATAT
TTTATATAATATTTTTTTATATAATACATTATAACATTAATATTATTTATATTATTTACATATTTTATTTTTAAA
AAACACATATAATTATAATAATTATAATAATAATAATTAAAATAATTTAATAAAATAAATATATATTTTAAATTA
TATATATTATAATATTTATTATATAAAAAAAATGTAATTACAAATTTATTTATACTAAAATTTATATTTATATTA
ATATAATTATCTTCTTTATAATTACATATTCTATACATATTATAATAAATAAAATATGTTAATAATATATTAATT
AAATAATATTTATTTTTACTAATATTTATATAAAAATTATTTAAATAGTTTTTATATATTAATCCTCTAAATATT
AAAGGATGTACTTTTTGTCCCATATCT

>Pf_ApiC | Plasmodium falciparum 3D7 | 514bp (n: 24167 - 24681)
Features: Geotyping sites api24426, api24456, & api24553

AACTACTCCTATAACTATATCACCATATTTAGGATTTTTATATTTATTTAAAGTACAAATATATTTAAATTTAAA
TATACCACTATTATCTATTACATCTAATATACTATTTATATATATCATAAAATTTTTATTACTTTATTATTACAA
TATTTACTTTTTTTATAATATTTAAATAATATATAATCATTTATAATAATTTCATTTCTATTATCATATATTTTT
ATATATAGATTACATAATAATAATTTTTTATATTTAAAATTATACTTATAAAAAGATATACAAACTATCTTAATA
TTTATATTTAAATTCTTAATAACATATCCTATTTTTATATTCATATTCTTTATTTAATTATTTTCTAATATATTG
TAATTTAAAAGGTAATTTATAAGATAAAACTTTTGTTATAGTATAAATAATATTATTTGAAATTTTACTTATTTC
AAATAATAACTTATTTTTTTTTATAGGACTTACATATAATTCTATAGATCCTTTTCCTGATCCCA

>Pf_ApiD | Plasmodium falciparum 3D7 | 1355bp (n: 22668 - 24022)
Features: Geotyping site api23716

TCTAATTCTTCCTTTTTTTATTGTATAAGATATTTTTTTTATTTCAATAATTTTTTCAATAATATTATTATTTAA
CTTTATAATACTATTATTTAACTTAATATTATTATTATATTTATAAATATAAAAATAAATAAAATTTACATTTTT
ATTATTAAAAATATATTTTTTATCTCTCTTATTATTTATAATATAATATAATTTTAAATATATAATTAAAATATT
ATAAATTTTATACAAATAATTATTAAACTTTCTTTTTAAAATTAATATAAAAATACTTTTAAATAATACTATAAC
ATAATATAATATATAAAAATTATTTAATTTTTTATTAATAATATTAATTATCATATTAATTTTTTTATACCTAAT
TCTTTATATTTATTTATATATTGAAATGAATTTATTAAATTCCCAATAGAATTTAATATAAATATATCCATACTA
TATAAATATATAAAATTTTTATTTATATCTAACTCACAAAATATTATATTAGGTAACTTAATAATTATTTTATGA
CTATATTTTAATTGAAATATTAAAAAATTACCTTTTTTTATATAATAAAATTTATAATTTATACCAATTATATTT
AAAATTAACTGATACTTTTTATATTGTATTTTATTAAATATATTACTAAATATATACTTATACATTTTAAATATA
AAATAATAAATATTATTTTTTATAAAATATATATATATATTATTTAATATATTAATATTATATATCTTATTATTA
TTTTTAAATATATATATATAATTCAAATATTTTATATCTAAAATTAAATATATATTACCTTCTTTTATATTATTA
TTTAAAAATATGTACTTTCTATTATTTATCATAACAATTATAACTATAATATTTACAAAATATAAAATAATAAAA
TACCACCAATTTTATATTTTAAAGCTAATATATGTGTAATAAATTTATATTTTGAAGAATATAAAATTAATAAAC
CTTTAAAATATTTTTTAAATTTTATAAAACATAATAATTTATTAAAATTCATATAAAATAATTGATTATATTTGA
TATATACTTTAAAATAATTAATATTTTTATATTCATTTAAAATTATAAAAATAAAATATTTATTATATATATTTA
AAATATATAATTTAAATATATATTTATAATTATATAATAATATACTTAAATAATATATTACTTTATTATATTTAT
ATAATATAAAATTTTTTTTTAATTTAAAATTATATTTAACATTATTTAAAAATTTAATAATCATAATTCAAATAA
ATTTATTTAAACTTATTCATATTTAATTTATATTTTATATATTTAGATATATAATTATTACTTTTAGTACCTACA
AAATT

>Pf_ApiE | Plasmodium falciparum 3D7 | 980bp (n: 22614 - 23593)
Features: Geotyping site api23070

TCCTAATCCTATCCAACCTTGTTTATTACCTAATACTAATACTATTTTATATCTTCTAATTCTTCCTTTTTTTAT
TGTATAAGATATTTTTTTTATTTCAATAATTTTTTCAATAATATTATTATTTAACTTTATAATACTATTATTTAA
CTTAATATTATTATTATATTTATAAATATAAAAATAAATAAAATTTACATTTTTATTATTAAAAATATATTTTTT
ATCTCTCTTATTATTTATAATATAATATAATTTTAAATATATAATTAAAATATTATAAATTTTATACAAATAATT
ATTAAACTTTCTTTTTAAAATTAATATAAAAATACTTTTAAATAATACTATAACATAATATAATATATAAAAATT
ATTTAATTTTTTATTAATAATATTAATTATCATATTAATTTTTTTATACCTAATTCTTTATATTTATTTATATAT
TGAAATGAATTTATTAAATTCCCAATAGAATTTAATATAAATATATCCATACTATATAAATATATAAAATTTTTA
TTTATATCTAACTCACAAAATATTATATTAGGTAACTTAATAATTATTTTATGACTATATTTTAATTGAAATATT
AAAAAATTACCTTTTTTTATATAATAAAATTTATAATTTATACCAATTATATTTAAAATTAACTGATACTTTTTA
TATTGTATTTTATTAAATATATTACTAAATATATACTTATACATTTTAAATATAAAATAATAAATATTATTTTTT
ATAAAATATATATATATATTATTTAATATATTAATATTATATATCTTATTATTATTTTTAAATATATATATATAA
TTCAAATATTTTATATCTAAAATTAAATATATATTACCTTCTTTTATATTATTATTTAAAAATATGTACTTTCTA
TTATTTATCATAACAATTATAACTATAATATTTACAAAATATAAAATAATAAAATACCACCAATTTTATATTTTA
AAGCT

>Pf_ApiF | Plasmodium falciparum 3D7 | 695bp (n: 21942 - 22636)
Features: Geotyping site api22599

ATCATTGTGTTTGCTTATGTTTTTTATTTATACAAATTATATGTAATTTTTTAAAACGTTTTATTAATTTACATT
TATTACATATTTTTTTTATTGAAGAACGTTTTTTCATTTTTTATATTAAATTTTTTTTATTTTATTTATAATTATA
TTATTTTTTTTTATTTTTAAAATTTTATGTAAATAAATATTTAATCTATATAAACTAATTTTAATATTACTGATA
TTATTTTCTTTAATATTAAATATACTTATATTATTTATCATTAATAATATATTAATATTATATAAATTTAACCCA
TATATATATTTTAATATATATATAAATAAATTTTTATTTAAATATAAAGTCATTTTAATTTTAAAATATATTAAA
TAATATTAATAATAATTTATTTAATAAATTATATCTATTATGTATTATATTATAATAAATTATTATTTTACAATT
AAAATAACCTAAACATTCAAATAAATATTTTAATAATAAAAATCTTATATTTAAATAATTATAAATTTTAAATTG
TAATTTAATAAAAAATTTACTATAATTAATATATATATATCTTAATTTATATATATTTAATATAGAATATTTAAA
ATAATAAATATTATTTAAAGCTTTTATTTTAGCAGATATAATAGCTTTATTAATATTGATATTCTTACTAACTCC
TAATCCTATCCAACCTTGTT

>Pf_ApiG | Plasmodium falciparum 3D7 | 953bp (n: 19673 - 20625)
Features: Geotyping sites api20428 & api20335

AAAGGTTTATGACGACCACCTTCTTCTTTAGTTAAAATATATGTTTCAGCTATAAAAGACTTATATACTTTTAAT
TTATTAGGTGTTGCTAAAATCATACCTCTTTTTATATCTTTTTTTTGAATATTTCTTAATAAAATACCTACATTA
TCTCCGGATTGTGCTTGTGTTAATTGTTTTTTAAACATTTCTAATCCTATAACTGTTGTTAAATTAGGAGATGAT
TTTTCAAATTTTAAAATTTCAATTTCATCATTTAAATTTATACATCCTTGTTCAATCTTACCTGTTACTACTGTA
CCTCTACCTGTTATAGAAAATACATCTTCTATTGACATTAAAAAGTAATCATTAATTTTTCTAGTAGGTATTATA
ATATTATCAATTATTTGAATTAAATTATTTAATTTTTGTATCCAAATATTAGATTTTATTAATTCATAATCCTTA
TTTTTTTGAATTATATTTATTACATTTAATGCTGAACCAGTTAATATATGTATATAATTTAAATCAAAATTATAT
TTAATTAATAATTCATTTACTTCTAATTTTATAAAATCTATTAATTCAACATCATCACATAAATCTTCTTTATTT
AAAAAAATAATTATATTTTTTATACCTATTTGTTTTATTAATAATAAATGTTCATAAGTTTGAGGCATTATACCA
TCTATTATAGATATTACTAAAATTGCTATATCCATTTGTGTGGCTCCTATAATCATATTTTTAATATAATCGGAA
TGTCCTGGACAATCTATATGAGCACAATGTTTTGTTAAAGTTTCATATTCAATATGTGTTGTATTTATTGTAATA
CCTCTTATTTTTTCTTCTGGAGCTGAATCAATATCTGAATAATTATATTTTTTTGATAATCCTTGTAAATTTAAT
AAATAAGATATAGCTGTTGTTAATGTAGTTTTTCCATGATCTACATGCCCTAT

```
>Pf_ApiH | Plasmodium falciparum 3D7 | 1023bp (n: 17895 - 18917)
      Features: Geotyping site api18365

ATTATCATTTACCCCTTCTAGTATTATATGTTTTTTTATTTTTAAATTTAAAATTTGTAATAACTGTATATATTG
TTGTTTATAAAAATTTAAATTATTATTATAAATATAATTTAAATTTAAAAGATTTACTGATATTTCTTTTAATTT
TATTCTAATATTATTTGAAAAAATATTAGTTTTTATATAATTATTTAAATTTAAATTAGAAAAATTTAAATTTAA
ATATTTAAATAAATAATTAATATCTTTATTATTATTTTTTTCTTCTAATAATAATAATAATAAATTAAATGAATT
AATTTTAAAATTAAAATTATTTAATTTAATTAATATATTAATAACTTTATTAGAAAAATTTATATTAATAATATT
TTTATTATTATATTTATATTTATTTAATAAAGATAAAATTATTTTATTATTTATTATTTTTTTATTTATTTTTAA
AAATTTTGTACATAAATTATCAGTTAATAATAATCCTAATAATAAATGAATAGGCATTATAAAATTATTATTATA
TTTTAATGCTAAATATTCAGATTTAATAAATATTATTATTAATTCTTTTGTACAATAAAGATTATTTAAAATTAT
CATGATTTTTATTATTTTTTATGTTTATTTGTTTAAAATTATTTTTTATAATATATAATAATTGATTAATATTAT
AAAATAATAATTGAAATTTTTTATATAATAATATCTTATATATTAGTAATATTTTATTTACTTTACTATTATATT
TATTATATAAATTATATATTGTATAATTATATATAATTTTAAATGTTTTGTCTGAATAAATATAAATTATAAATA
AATTCAATTTATATTTATTTATATATTCTGTTATTTTTTTATTTAATAAATTAATATTAATGTTATATATTTTAA
ATTTAAAATTTAAAATTGAATTTAAATTTATTTGATTATTTTTTATTAAAAATTTATATTTACATGAAATAATCA
TTTTTTTATTTATATATTATTATATACATGTCCTAAAGGATTCGAACC
```

```
>Pf_ApiI | Plasmodium falciparum 3D7 | 932bp (n: 16998 - 17929)
      Features: Geotyping sites api17812 & api17760

ACCACTAGGCCCACATAAAATCCAACTTCCTATAGGTTTATTTTTATTTTTTAATCCTATAAAATTTTGTTTAAT
AAAAGGTATTATTTTATTAAAAATATGATTTTGACCATATATATGATTATATAAATAATTTTCTAATTTAGTTAA
ATTTAATTTAGTTAATTTATTGTCTTTAAATAATATGGTTTTAGATATATTTAAGTAATTAGATATTGAATTTTT
AATATCTTCTATAGTTAAATTATTATTTTTATTATTATATATAATATTATTATTATATGTAAATAAATAATTAAA
ATTTGAATAAGATATTTTATTATTTAATAAATATTTATTAGAACATGAATTTTCTAATAAAATTAATGGAGTTGT
AGGTAATATTAAAGGTTTTATATACTTTTTACTTAAATTTATTAATTCATATATAATATAATTATTAATATTAAT
TTTATAATAATTAATATATTTATATATATTATTTTTTATTATTAAGAATGTTTGTAATATTGATAAATCTTTTAT
TCTTATTTTATAAAAATAAGAATCTTTTATTATATTATATTTAAAATATGTATTATATTCTTTTTTATTAGTTAC
TATTATTATATGTATATTATATCCATATAATTTATTTAATAATAAAAATAAATAATATAATTTATTATTATCATT
ATTAATATTATCTGATAGATTAAATATTTCTATATTTTTTATAATTAAGATTAATTTATATTTATTTGTAAAATA
TTTAGATATATTCAATATTTTATATATTAAAGTTTGTATATCATAAGTTAATAAATCATTTAATACCCATATTTC
TGTATATTTTAAATATATTGGTATAATTTTATTTTTTATATTATTAATTAATAATTGTAAAAATATAAAAATATT
ATCATTTACCCCTTCTAGTATTATATGTTTTT
```

```
>Pf_ApiJ | Plasmodium falciparum 3D7 | 439bp (n: 14267 - 14705)
      Features: Geotyping site api14300

ATTTATCAAGCAACTAAAAGTTCATATATATATGTATATAATATTTTAATTGAATCTATTTTGAAACAATATAGT
TATCAAAATATTTATTTACCTTCTATTTATTTTGAACTTATTATAAAAAAAATGTTATCATGTATAAAGATAATA
TCTAATAATTTTAAAATATTTAAATATAATGATATAATATCTTTACAATTAATAAATATAATTAATTATTCTTTA
AATTTAAATAAACATTATATTTATAAATATGAACCAATTATTTTAGGTATTACAAAATCAATTTTAGCTAATTCT
GGATTTTTAACAAATATAAGTTTTCAGAATACATTTAAAATAATAAGTTTAAATATTTTAAATAATAAAATTGAT
TGGTTAATAGATATAAAATCTAAAATAATATTGACTGATTTATTACCTGTTGGTAATGGATGGT
```

>Pf_ApiK | Plasmodium falciparum 3D7 | 613bp (n: 8159 - 8771)
Features: Geotyping sites api8600 & api8243

GAAGGATTAACTTGTGGTTTAGTTAATTATTTAACTACAAATATATTTTTAAATTTAAAATATTTATTCGTAATA
TATTATAAACATATATTTTATAATAGATATAATTTTAAGTTATTATTAAATATATTTAATAAAAATTTTTATAAT
ATTAGTTTTAATAATATTTATTTAAAAAAAAATATTAATTTTAATAAAACAACTATTTTAACAATAAATAAAAAT
ACATTTAAAATATGTAATATAACACAAAATATTATTTACATACCTTTTAATTATTTATTATCTTTTATTGAAAAT
TTAATACCATTTATACATTATAATGATTCTATTAGAAATTTAATGAGTATAAAAATGCATACTCAAATTGTTCCT
ATTATATATCCAAATTTAAGTAATATTATTACTAATTATAATTTTATTTTAAATAAATATTTAAATCATTTAATT
ATTTCATATCAAGAAGGAATAGTTATATATGTTTCTTGTATAAAAATAATAATAAGAGATTTATTTAATAGACAA
ATAATTTATTATTTAAATAATTATAAAAAAATAAATCAAAATATATTATTAATTTATAAACCTATTGTATGGGTA
GGGGAAAAAGTTA

>Pf_ApiL | Plasmodium falciparum 3D7 | 944 bp (n: 5095 - 6038)
Features: Geotyping site api5628

AGTTCAAATCTCACCATTAGCTTTTATTATTTTTATATATAATTATTATGATAAAATTAAAAAATTTTTTAAATA
TTTATAATTTAAATTATAAATATCAATATAAAAATAAAATAAATTTATATTTAATAAGACAAGGATTAAATATAA
ATTTAATAAAAAATTTATCTAGTAATATTTTTTTATATATGTTTATTTATAATTTTAAAAAATATTCTTTAAAAT
TATTAAATATATTTAAATTACCTGATTGGAATTTTTTTGATTGTCCAAATATAAATTATGATAATATTATTTATT
ATTCTTCTATTTTAAAAGATAATAATTTAATATATTATTTAAAAAATAATTTAAATATTGAATTTTTAGATAGTA
TATTAATAAAAAATAATTCTATAGATATTATATTTGATAGTATGTCTATTTTACATACTACACAATATTTTTTAA
AAAAATTAGGAATAATTTTTTTACCTTTATTTGATATTATATTTAAATATCCTTTATTAATAAAAAAATATTTAG
GTACTATTATTTCTTATAAAGATAATTTTTTTGCTAATATTAATTCAATAATATTTAGTGAAGGATCTTTTTGTT
ATATACCTAAATATGTAAAGTGTAATTTTAATTTATCAACATATTTTAAAACTAATTCTTCTGATTTTGCACAAT
TTGAACGTACTTTAATAATAGTTGGTAAATATTCTTATGTATCATATTTAGAAGGATGTACAGCTTCATTATATA
AAGAATCACAATTACATGTAGCTATAGTAGAAATAATAGTAAAAGATTATGGTTATATAAAATATTATACATTAC
AAAATTGGTATAGAGGAGATTATTTAGGTAATGGTGGTTTATATAATTTTACAACTAAACGTGGTATATGTTTAA
ATTATTCAAAATTAGATTGGATACAAGTTGAAGTAGGTTCGATT

>Pf_ApiM | Plasmodium falciparum 3D7 | 814 bp (n: 2271 - 3084)
Features: Geotyping site api2772

GGTTCAATTCCGTTTTTCGCTTATAATTATATAAATTTATTTATTATAATTTAAATTAAATATATAATTAAATAT
ATATAATAAATTTAAAAGGATGTCTATAATTTTTATTAAATAAGAAAGACGTTATATATATTAACGAAATTTTTA
TAATAATTAATAAAAATATATTTATATTTATTAATAAATATTATTATAAAAGTATCTGATAAAATATTTTTATAT
AAATATTTTATTTAAAAACTTAAGGAATTGAATCATCTTAGTACTTAAAGGAATAAAAAGTAATAACGATTTTCA
TAGTAGCGGCGAGCGAATTGAAAAAAAATAAACATTTAAAAAAATATGTAAAATTTTAATAAAGTAATATAATAT
ATAATTTTATATAAATAATATAAAAAATAATAAGAATTACCTTATAAAATTAAATATAATAAAAGTTTATAGAGA
AAAGTACCGTGAGGAAAGATGAAAAGAATTTTGAAAAAATAGTGAAAAGAATCTGAATTTTAATTTAAATAAATT
ATAATAAAATTTAATTTTAATTTTATTATATTCTTGTTGAAGAAAGAATTAGCAAGTTATAATAAATAGTATAAT
AATTAGGTTAAATATATTTAAATAATGAAGCCAAAGTTAAATCGAATTTATTTTTTTAATAAATTTAAAATATAT
TATTTATTATAGACCCGAAATCAAATGATCTAATTATATTTAAAATAAAATTTAAATAAAATTAAATTATAGTTT
GAATTGATTACTGTTGCAAAAGTATCAAATAAAATATAATTAGTGGTGAAATGCCAATCGAATT
